# Drastic reduction of false positive species in samples of insects by intersecting the default output of two popular metagenomic classifiers

**DOI:** 10.1371/journal.pone.0275790

**Published:** 2022-10-25

**Authors:** Lidia Garrido-Sanz, Miquel Àngel Senar, Josep Piñol

**Affiliations:** 1 Universitat Autònoma de Barcelona, Cerdanyola del Vallès, Spain; 2 CREAF, Cerdanyola del Vallès, Spain; University of Helsinki: Helsingin Yliopisto, FINLAND

## Abstract

The use of high-throughput sequencing to recover short DNA reads of many species has been widely applied on biodiversity studies, either as amplicon metabarcoding or shotgun metagenomics. These reads are assigned to taxa using classifiers. However, for different reasons, the results often contain many false positives. Here we focus on the reduction of false positive species attributable to the classifiers. We benchmarked two popular classifiers, BLASTn followed by MEGAN6 (BM) and Kraken2 (K2), to analyse shotgun sequenced artificial single-species samples of insects. To reduce the number of misclassified reads, we combined the output of the two classifiers in two different ways: (1) by keeping only the reads that were attributed to the same species by both classifiers (intersection approach); and (2) by keeping the reads assigned to some species by any classifier (union approach). In addition, we applied an analytical detection limit to further reduce the number of false positives species. As expected, both metagenomic classifiers used with default parameters generated an unacceptably high number of misidentified species (tens with BM, hundreds with K2). The false positive species were not necessarily phylogenetically close, as some of them belonged to different orders of insects. The union approach failed to reduce the number of false positives, but the intersection approach got rid of most of them. The addition of an analytic detection limit of 0.001 further reduced the number to *ca*. 0.5 false positive species per sample. The misidentification of species by most classifiers hampers the confidence of the DNA-based methods for assessing the biodiversity of biological samples. Our approach to alleviate the problem is straightforward and significantly reduced the number of reported false positive species.

## Introduction

High-throughput DNA-sequencing (HTS) technology has revolutionised the assessment of biodiversity in biological communities. The process produces millions of reads of many species from one or a few genomic regions (in metabarcoding) [1] or from the entire genome (in shotgun metagenomics) [2]. These reads are then compared to DNA sequences from genetic repositories to obtain taxonomic information.

The bioinformatic tools and pipelines used to assign HTS reads to species come with many names, but we refer to them here generically as metagenomic classifiers. There is a myriad of such tools [3, 4], but considering only the classifiers that assign individual query sequences to reference sequences by similarity, there are two general strategies in a compromise between accurate results and fast execution times. Tools specifically designed to provide highly precise classification by the alignment of reads against reference sequences and return the most similar matches. Despite significant improvements in aligners performance, this approach is computationally intensive. Popular tools of this group are BLAST [5], Bowtie2 [6] and BWA [7]. Alternatively, classifiers can reduce the complexity of the alignment at the expense of sensitivity. A very efficient strategy is based on *k*-mers (sub-strings of length *k*); rather than mapping the whole read, the *k*-mers of a query read are directly associated with reference taxa that contain the same *k*-mers. Examples of classifiers of this group are Kraken [8], CLARK [9] and Kallisto [10]. In both cases (whole-read alignment and *k*-mer-based) several taxa can be associated with a read, so an algorithm is needed to assign a taxon to each read; the most common approach is the so called lowest-common ancestor (LCA) algorithm, implemented, among many others, in MEGAN [11].

When the metagenomic classifiers are used with the default parameters in samples of known composition, they normally produce a high number of false positives species (*i*.*e*., they detect species that are not present in the sample) [12, 13]. This is highly problematic and reduces the reliability of DNA-based methods to describe the biodiversity of complex biological samples where the prior composition is unknown. In such circumstances, a critical mind cannot stop wondering whether she is watching a highly diverse sample or an artefact of the metagenomic classifier. To reduce the number of false positives there are two main approaches. (1) The filtering or post-processing of the classifier’s output to refine the assignment [11, 14–17] and (2) the simultaneous use of several metagenomic classifiers that independently assess each sample and produce a combined result [18–20].

Here we tested two popular metagenomic classifiers (BLASTn [5] followed by MEGAN6 [21]; and Kraken2 [22]) using their default parameters against a set of shotgun sequenced reads from insect species. As expected, both metagenomic classifiers produced a good deal of false positive species. To reduce their number, we combined the results of the two classifiers in two simple ways that we call union and intersection. In both approaches when a read *r* was assigned to a different species by each classifier, the read *r* was discarded; however, the two approaches differed in the way that a read *r* was treated when assigned to some species *s* by one method but not assigned to any species by the other one. In the union approach, the read *r* would be assigned to species *s*. On the contrary, in the intersection approach such a read *r* would not be assigned to any species (Table 1). The intersection method is more restrictive as it only keeps the reads assigned to the same species by the two classifiers, whereas the union method intends to extract as many informative reads as possible from the sample.

**Table 1:** Rules of classification of a read *r* using the union and the interception approaches of two metagenomic classifiers *p* and *q*. The read *r* can be assigned to a species (*e*.*g*., species *s* or species *n*) or can remain not assigned (*NA*).

Case	Classification of read *r* by		Classification of read *r* when merging results with	
	Classifier *p*	Classifier *q*	Union	Intersection
#1	*NA*	*NA*	*NA*	*NA*
#2	Species *s*	*NA*	Species *s*	*NA*
#3	*NA*	Species *s*	Species *s*	*NA*
#4	Species *s*	Species *n*	*NA*	*NA*
#5	Species *n*	Species *s*	*NA*	*NA*
#6	Species *s*	Species *s*	Species *s*	Species *s*

The objective of this study is to reduce the production of false positive species by metagenomic classifiers using simple, straightforward methods. (1) We use the classifiers with their default parameters; we are aware that this is not always advisable, but in general it is difficult to tune the parameters without a lengthy calibration process. Besides, most applications do use the default parameters of the tools (*e*.*g*., [12, 23, 24]). (2) As the metagenomic classifiers with default parameters produce a high number of false positive species, we combine two of such classifiers with the union and the intersection strategies outlined above to reduce the number of false positive species. (3) Finally, we refine the results by establishing an analytic detection limit to reduce the number of reported species reported. We test our methods using several single-species DNA samples from a previous study [15]. Arguably, this kind of sample with only one species is especially suited for the test, because we know for every read the species to which it belongs; on the contrary, in artificial samples of several species, it is known the relative proportion of every species, but not the identity of every read. The metagenomic classifiers compared the obtained reads of the single-species samples with a reference database of *ca*. 2000 mitochondrial genomes of insects, in what is called mitochondrial metagenomics [25].

## Results

### Individual classifiers

The use of the two metagenomic classifiers with the default parameters detected a high number of species in the single-species libraries, where in theory there should have been only one. The BLASTn followed by MEGAN6 method (BM) produced 13.2 ± 7.7 species per sample (Table 2A) belonging to 11.0 ± 7.7 families and 5.0 ± 2.4 orders per sample (S1 Table). Kraken2 (K2) produced an even higher value of 321.7 ± 122.7 species per sample belonging to 142.1 ± 38.3 families and 21.9 ± 3.0 orders per sample. The precision (see Material and methods) was higher for BM than for K2 (0.986 ± 0.015 *versus* 0.757 ± 0.127) (Table 2A). As only the reads mapping into the mitogenome are useful in mitochondrial metagenomics [16], both classifiers used a very low proportion of reads (RPIR; see Material and methods) (BM: 0.0069 ± 0.0069; K2: 0.0063 ± 0.0056). The recall (see Material and methods) was also higher with BM than with K2 (0.864 ± 0.158 *versus* 0.820 ± 0.097) (Table 2A and S2 Table). Finally, K2 was *ca*. 60 times faster than BM (Table 2A).

**Table 2:** Summary table of metrics results of the methods for species identification. Benchmark metrics scores for each classifier without detection limit (A), with an analytical detection limit of 0.0001 (B), and with an analytical detection limit of 0.001 (C). For richness, the relative proportion of informative reads (RPIR), precision and recall we provide the mean and standard deviation of all 21 samples; for the processing time we provide the sum of the total consumed time when running all the samples sequentially (format hh:mm:ss). The time for creating the databases and running in-house python scripts are omitted.

(A) Metric	BM	K2	Union	Intersection
Richness	13.2 ± 7.7	321.7 ± 122.7	316.7 ± 122.4	2.3 ± 1.9
RPIR	0.0069 ± 0.0069	0.0063 ± 0.0056	0.0072 ± 0.0065	0.0055 ± 0.0054
Precision	0.986 ± 0.015	0.757 ± 0.127	0.822 ± 0.098	0.998 ± 0.005
Recall	0.864 ± 0.158	0.820 ± 0.097	0.926 ± 0.053	0.684 ± 0.174
Processing time	01:51:30	00:01:55	01:52:42	
(B) Metric	BM	K2	Union	Intersection
Richness	13.0 ± 8.0	232.0 ± 129.8	215.1 ± 122.4	2.1 ± 1.5
RPIR	0.0069 ± 0.0069	0.0063 ± 0.0056	0.0071 ± 0.0065	0.0055 ± 0.0054
Precision	0.986 ± 0.015	0.762 ± 0.131	0.827 ± 0.103	0.998 ± 0.005
Recall	0.864 ± 0.158	0.82 ± 0.097	0.926 ± 0.053	0.684 ± 0.174
(C) Metric	BM	K2	Union	Intersection
Richness	4.2 ± 3.6	36.5 ± 38.1	32.1 ± 38.2	1.5 ± 1.1
RPIR	0.0069 ± 0.0069	0.0063 ± 0.0056	0.0071 ± 0.0065	0.0055 ± 0.0054
Precision	0.989 ± 0.015	0.806 ± 0.141	0.872 ± 0.105	0.998 ± 0.005
Recall	0.864 ± 0.158	0.820 ± 0.097	0.926 ± 0.053	0.684 ± 0.174

There were also reads that could be genuinely attributed to contamination, both from the lab and from the field sampling. The reads assigned to contaminant species are neither reported in the above results nor in Tables 2 and 3, but they are provided as supplementary material (S1 Table). More details about the contaminant species are provided in the Material and Methods section and in [15].

**Table 3:** False positive species detected on each library by the intersection approach. For each library, we indicated the run and library codes, the name of focal species (its order in brackets), the number of congeneric species in the reference database, and a list of the false positive species divided in congeneric and non-congeneric to the focal species. The last three columns contain the number of false positive species detected with the analytical detection limits (ε) of 0, 0.0001 and 0.001. For each non-congeneric species to the focal species, we indicated, in brackets, the RPIR and its order. Order abbreviations are Col: Coleoptera, Dip: Diptera, Hem: Hemiptera, Hym: Hymenoptera, Lep: Lepidoptera.

Run—Library	Name of the focal species (Order)	Num. congeneric species within database	False positive species		Number of false positive species		
			Congeneric to focal species	Non-congeneric to focal species	ε = 0	ε = 0.0001	ε = 0.001
1–1	*Papilio machaon* (Lep)	14			0	0	0
1–3	*Drosophila melanogaster* (Dip)	20			0	0	0
1–4	*Drosophila mojavensis* (Dip)	20			0	0	0
1–6	*Linepithema humile* (Hym)	0			0	0	0
1–9	*Acyrthosiphon pisum* (Hem)	0			0	0	0
2–1	*Atta colombica* (Hym)	0			0	0	0
2–4	*Drosophila melanogaster* (Dip)	20			0	0	0
2–5	*Drosophila mojavensis* (Dip)	20			0	0	0
2–7	*Drosophila suzukii* (Dip)	20			0	0	0
2–8	*Linepithema humile* (Hym)	0			0	0	0
2–11	*Vollenhovia emeryi* (Hym)	0			0	0	0
1–2	*Drosophila virilis* (Dip)	20	*D*. *littoralis* (0.0008)		2	1	0
			*D*. *incompta* (<0.0001)				
1–5	*Bactrocera oleae* (Dip)	14	*B*. *biguttula* (0.0018)		1	1	1
1–7	*Bombus terrestris* (Hym)	3	*B*. *hypocrita* (0.0035)		3	3	3
			*B*. *waltoni* (0.0023)				
			*B*. *ignitus* (0.0015)				
1–8	*Apis mellifera* (Hym)	7	*A*. *nigrocincta* (0.0011)		7	4	1
			*A*. *florea* (0.0006)				
			*A*. *laboriosa* (0.0002)				
			*A*.*nuluensis* (0.0002)				
			*A*.*andreniformis* (<0.0001)				
			*A*. *cerana* (<0.0001)				
			*A*. *dorsata* (<0.0001)				
2–3	*Cimex lectularius* (Hem)	0		*Reduvius tenebrosus* (Hem) (0.0009)	2	2	0
				*Aquatica wuhana* (Col) (0.0001)			
2–9	*Plutella xylostella* (Lep)	1		*Prismognathus prossi* (Col) (0.0002)	1	1	0
2–12	*Wasmannia auropunctata* (Hym)	0		*Vespa orientalis* (Hem) (0.0028)	4	4	3
				*Eriogyna pyretorum* (Lep) (0.0013)			
				*Pristomyrmex punctatus* (Hym) (0.001)			
				*Allocarsidara bakeri* (Hem) (0.0001)			
2–2	*Bemisia tabaci* (Hem)	1	*B*. *afer* (0.0008)	*Barca bicolor* (Lep) (0.0004)	3	3	0
				*Trialeurodes vaporariorum* (Hem) (0.0004)			
2–6	*Drosophila virilis* (Dip)	20	*D*. *littoralis* (0.0003)	*Pachycerina decemlineata* (Dip) (0.0002)	2	2	0
2–10	*Solenopsis invicta* (Hym)	2	*S*. *richteri* (0.0183)	*Myrmica scabrinodis* (Hym) (0.0013)	3	3	3
			*S*. *geminata* (0.0039)				

### Combined classifiers

The combination of the outputs of the two classifiers with the union method still produced a richness much higher than expected (317 ± 122 species per sample) (Table 2A). This value is just slightly lower than the one produced by K2 alone, so the union did not help to get rid of false positive species.

On the contrary, the combination of the outputs of the two classifiers with the intersection method drastically reduced the number of false positive species. The recovered richness decreased to 2.3 ± 1.9 species per sample and the precision was also much higher (0.998 ± 0.005) (Table 2A). In fact, there were no false positive species in 11 samples (out of 21) (Table 3). In 4 of the remaining samples, the false positives species were of the same genus to the focal species, whereas in the last 6 samples there were species of a different genus or even of a different order (Table 3 and S1 Table). On the negative side, the elimination of reads reduced the RPIR to 0.0055 ± 0.0054 (Table 2A).

### Use of an analytical detection limit

The use of an analytical detection limit of 0.0001 (0.01%) slightly reduced the number of detected species (Table 2B). The more stringent detection limit of 0.001 (0.1%) removed many more false positives species (Table 2C). Indeed, the combined use of the 0.001 detection limit with the intersection approach reduced the number of recovered species per sample to 1.5 ± 1.1 (16, out of 21, samples were free from false positive species; Table 3) (results of all libraries and methods are provided in S1 and S2 Tables).

## Discussion

The occurrence of false positive species in shotgun sequenced DNA samples seems to be a universal feature that compromises the reliability of the method. Whereas some false positive species are produced by contamination during the sampling, in the lab or during the sequencing [26–28], many others are produced by the bioinformatic tools used to assign species to reads [29–31]. In this study, we have examples of both kinds, but we were able to identify the contaminant species because we knew which ones were handled simultaneously in the lab. Regarding the misclassifications caused by the bioinformatic tools, we were able to almost eliminate all misidentified species by post-processing the output from two popular metagenomic classifiers in a very simple way.

### Individual metagenomic classifiers with default parameters

In the literature, different metagenomic classifiers have been compared against each other many times to seek the most suited one depending on the characteristics of the target organisms, laboratory treatment, sequencing technologies, read length, taxonomic rank, database completeness, etc. [17, 23, 29, 30, 32]. Our approach using the individual classifiers produced results like those reported in the literature. Thus, studies running BLAST, with or without MEGAN, had a precision above 90% [14, 19, 23, 33], as we found here. Similarly, the precision reported with Kraken2 is lower, 75–85% [19, 34, 35]; again, these results are in concordance with our findings. Other studies also report a very long list of false positive species for Kraken2 [34, 36] as we did here (S1 Table).

The reasons that explain why some methods work better for a particular kind of sample (*e*.*g*., BM works better than K2 with our insect samples) depend on many factors [12, 19, 33, 36]. One plausible explanation is that the alignment of whole-reads is far more complex than matching multiple *k*-mers so it provides more robust identifications [37]. A second explanation is that both methods have options to refine their result, but Kraken2 does not use them by default (*e*.*g*., Kraken2 has a *confidence* score with a default value of zero, so most reads are assigned even when the confidence is very low). On the contrary, BLASTn and MEGAN6 apply filtering options (e.g., bit-score, e-value, top percentage, minimum score) that help reducing to some extent the number of false positives. These options can be easily tuned, and the only way to set the proper thresholds is by calibrating the tool with samples of known composition.

### Combination of the two metagenomic classifiers

The fact that the intersection method significantly reduced the number of false positive species suggests that different classifiers misidentify reads in different ways. Consequently, the most robust way to present the results is to keep as informative only the reads assigned to the same species by the two classifiers. The important reduction in false positive species is accompanied by a modest reduction of false positive reads, as most false positive species were represented by a low number of reads.

The metagenomic classifiers are not able to deal with all the genomic peculiarities of every species on Earth [33, 37]. Thus, certain classification strategies may perform better on particular scenarios. For example, alignment methods like BLASTn provide better results when databases are large and highly heterogenous [31]; whereas *k-mer*-based methods like Kraken2 may be better suited for species with frequent structural changes [33]. Our results showed that metagenomic classifiers with different identification strategies rarely misassign a given read to the same (wrong) species. So, the intersection method, albeit conservative, double checks the association of reads to species to ensure confident identifications.

There are several other tools devised to unify results from several classifiers but, in general, they are more complex or require the use of specific software. These tools either combine profiling (*e*.*g*., MetaMeta [24] merges six tools) or read-a-read assignments (*e*.*g*., WEVOTE [38] combines five tools by default and PhymmBL [39] combines Phymm and BLAST). The strategies used to merge tools can vary widely, but they generally infer taxa with a voting system or rank taxa with probabilistic scores. In general, these tools show that precision is higher when multiple classifiers are combined. Similarly, McIntyre et al. [19] applied various ensemble approaches (*e*.*g*., maximum-voting and abundance ranking) that outperformed individual tools. In terms of precision, our results were similar or even better than those reported by more complex methods [19, 24, 38], but much simpler to understand and to implement.

### The use of an analytic detection limit

The number of false positive species could be further reduced by using a threshold below which the occurrence of a species in the species list of a sample is ignored. As noted above, false positive species generally have a low number of assigned reads, so the use of a simple threshold or detection limit reduced the number of false positive species without losing many reads. This approach is by no means new, as many authors use a detection limit to get rid of species, either in absolute terms (species must be above a certain number of reads) or in relative terms, as we do here [17, 40, 41].

There are other methods to discard unwanted species that have not been considered here, like the analysis of the distribution of reads across the genome [42], the calibration or tuning of the parameters of the metagenomic classifier [20, 31], the replication of samples [26, 43], the use of negative controls [27, 43–45], the removal of low complexity sequences [16, 24, 46], cleaning reference database from contaminants [15, 46], limiting the reference database to target species or sequences [47–49] or removing false positive species that are unlikely present in the sample [27]. All these methods would probably further reduce the number of false positive species but at the cost of a more lengthy or more expensive process.

## Conclusions

DNA-based identification methods based on HTS holds great potential for the study of biodiversity and interactions in ecological communities, yet this approach is not free from shortcomings. One important of such shortcomings is the ubiquitous false positive species produced by most metagenomic classifiers [13]. Unless we find ways to reduce the number of false positives in samples of known composition there will always be a shadow of a doubt about the high diversity reported in many field studies [50]. Here we showed that the simple intersection of the output of two very different metagenomic classifiers drastically reduced the number of false positives. When this result was combined with the application of an analytic detection limit of 0.001 (*i*.*e*., species below an abundance of 0.1% are not considered), the number of false positive species was reduced to a manageable figure of *ca*. 0.5 false positive species per sample. All this was accomplished using the default parameters of the two classifiers, making our approach extremely straightforward and at reach to most research labs, even to those without strong bioinformatic expertise.

## Material and methods

The libraries of insect’s DNA used here to benchmark the metagenomic classifiers were created in a previous application of whole-genome and mitochondrial metagenomics for the classification of species with sequenced genomes [15, 16] (the sequenced libraries are available at DRYAD repository [51]). Despite using the data of previous studies, the present one is fully independent. Instead of assessing the capabilities of whole-genome and mitochondrial genome for the classification of species of insects as we did before, here we compare the performance of popular bioinformatic tools, like BLASTn [5], MEGAN6 [21] and Kraken2 [22], that we did not use before. Below we provide a short explanation on data gathering, but for more detailed information the reader is referred to the parent studies.

### Reference mitogenomes

We downloaded all mitogenomes of insect species available on RefSeq repository plus 11 mitogenomes from GenBank of insect species whose complete genomes were available on RefSeq but that their mitogenomes were not (both repositories were consulted on 3rd May 2020). Species with more than one mitogenome were randomly dereplicated. We obtained a total of 1934 mitogenomes (S3 Table).

### Preparation of samples: Selection of species, laboratory, and quality control

There were 21 single-species libraries, each one of them containing DNA of one insect species whose mitogenome was available in the reference database. There was a total of 17 species, as 4 species were sequenced twice in different runs. Illumina MiSeq technology was used to shotgun sequencing at 2x150 bp, albeit we only used here the forward read of the pair (R1 files, available via [51]) because many real eDNA samples are likely to have very fragmented DNA. The single-end reads were quality filtered with FastQC [52] and Trimmomatic [53] (minimum length of 140 bp and cropped at 150 bp).

### Classification of reads to species

#### Individual classifiers

We selected two pipelines that assign DNA reads to species to independently identify the insect species within the single-species samples, (1) BLASTn [5] followed by MEGAN6 [21] (BM) and (2) Kraken2 [22] (K2). These tools were chosen because they are widespread among the bioinformatic community and because the underlying algorithms belong to very different approaches. Briefly, BLASTn search for similarities between the query and the reference sequences with local alignments from short exact matches and then extends the alignment to the rest of the query sequence (seed-and-extension algorithm); as multiple matches are reported, MEGAN6 is subsequently used to assign the query reads to taxa using the lowest-common ancestor (LCA) algorithm. On the contrary, Kraken2 seeks for exact matchings between the read’s *k*-mers and reference taxa *k-*mers; then, it uses an LCA algorithm to assign a taxon to each read. As we are only interested in the classification at the species level, we ignored in both classifiers the assignments to superior levels of taxa.

Some of the species reported by the metagenomic classifiers in our samples can be genuinely attributed to cross-contamination, either from the laboratory (*i*.*e*., species sequenced on the same sequencing run), or from the field sampling (*i*.*e*., species trapped together). As we know the identity of the contaminant species, we eliminated them from the analysis. A more thorough explanation of the contaminant species is provided elsewhere [15].

#### Combination of results: Union and intersection of classifiers’ results

We combined the results from BM and K2 in a single common output in two distinct ways: union and intersection. In the union approach, a read is assigned to the species identified by any classifier unless both classifiers assign the read to different species, in which case it is discarded (Table 1). Thus, the union approach reduces the number of informative reads by eliminating those assigned to different species by the two pipelines. However, it also increases the number of informative reads by keeping those assigned to a species by any classifier, even if the other one did not assign the read. The intersection approach is much more restrictive, as only assign a read to a species when both BM and K2 provide the same result (Table 1).

#### Analytical detection limit

We further refined the above results (both from individual classifiers and the combination of results) by using an analytic detection limit. Thus, to include a species in the species list of a sample, its abundance must be above the threshold or detection limit. We report the results without a detection limit and with the detection limits of 0.0001 (0.01%) and 0.001 (0.1%).

### Tool commands

#### BLASTn + MEGAN6 classifier

For BLASTn [5], all mitogenomes were stored in a single file (1934mitogenomes.fna) and a database (1934mitogenomes.db) was build using the following commands. Sequences downloaded from RefSeq and GenBank had to change their sequence’s ID and include the taxon ID to work with MEGAN6 [21]. For example, the header for the mitogenome of *Drosophila melanogaster* was “>Drosophila_melanogaster_taxid_7227”. All sequences’ ID along with their taxon ID were stored in taxid.txt file. This file is required during the database construction of BLASTn.

*$ makeblastdb -in 1934mitogenomes.fna -parse_seqids -blastdb_version 5 -taxid_map taxid.txt -title "1934mitogenomes.db" -out 1934mitogenomes.db -dbtype nucl*

We subsequently matched samples (query.fasta) to reference mitogenomes with BLAST’s *blastn* and assign them to taxa using the LCA algorithm with MEGAN6’s *blast2rma*.

*$blastn -db 1934mitogenomes.db -query query.fasta*

*-num_alignments 10 -out BLASTn.tab -outfmt 6 -num_threads 12*

*$blast2rma -f BlastTab -bm BlastN -alg naive -i BLASTn.tab -o MEGAN.rma*

The output of the assignment is provided in two different formats, as classes counts (-c2c option) and read to class assignment (-r2c option), and using the scientific name and taxon ID.

*$rma2info -c2c Taxonomy -r -n True -i MEGAN.rma > MEGAN_c2c_sciNames.txt*

*$rma2info -c2c Taxonomy -r -n False -i MEGAN.rma > MEGAN_c2c_taxID.txt*

*$rma2info -r2c Taxonomy -n False -i MEGAN.rma > MEGAN_r2c_taxID.txt*

We then retained only the assignments at the species level with in-house python scripts. Notice that we did not use the “-r” option that reports the taxonomic rank of the classification with “rma2info -r2c Taxonomy” command because “group” (*i*.*e*., melanogaster group) is denoted as species “S” when it is not, yet with “rma2info -c2c Taxonomy” command is no-ranked with “-”.

*$python3.7 MEGAN_LCACounts2SppCounts.py -i MEGAN_c2c_sciNames.txt -o MEGAN_c2c_sp.txt*

*$python3.7 MEGAN_LCAReads2SppReads.py -i MEGAN_r2c_taxID.txt -t MEGAN_c2c_taxID.txt -o MEGAN_r2c_spTaxID.txt*

The output containing read counts (MEGAN_c2c_sp.txt) was used to evaluate the results of the BM method when this classifier was used alone, whereas the output containing the classification of the reads (MEGAN_r2c_spTaxID.txt) was used for combining the results from the two classifiers.

#### Kraken2 classifier

We built a custom database for Kraken2 [22] using the commands indicated below. We named the Kraken2’s database Kraken2_DB and the path to the folder containing all 1934 mitogenomes as MITO_PATH. The headers of the mitogenomes were modified to fit the sequence ID custom for Kraken2. Sequence ID structure was “>NNNN|kraken:taxid|XXXX”, where NNNN and XXXX are replaced by the accession number and species taxon ID code from NCBI, respectively. For example, the header of the mitogenome of *Drosophila melanogaster* was “>NC_024511.2|kraken:taxid|7227”.

*$ kraken2-build—download-taxonomy—use-ftp—db Kraken2_DB—threads 12*

$for mitogenome in MITO_PATH/*fna; do kraken2-build—threads 12—add-to-library $mitogenome—db Kraken2_DB; done

*$kraken2-build—build—threads 12—db Kraken2_DB*

FASTQ samples (query.fastq) were subsequently classified and saved the reads assignment with scientific names (K2_r2lca_sciNames.txt) and taxon ID (K2_r2lca_taxID.txt); and also the summary report (K2_report.txt).

*$kraken2—db Kraken2_DB—threads 12—use-names—output K2_r2lca_sciNames.txt—report K2_report.txt query.fastq*

*$kraken2—db Kraken2_DB—threads 12—output K2_r2lca_taxID.txt query.fastq*

Then, we retained only the assignments at the species level using in-house python scripts.

*$python3.7 KRAKEN_report2SppCount.py -i K2_report.txt -o K2_report_sp.txt*

*$python3.7 KRAKEN_LCAReads2SppReads.py -t K2_report.txt -i K2_r2lca_taxID.txt -o K2_r2lca_spTaxID.txt*

Finally, an output file containing species-level counts (K2_report_sp.txt) was used for assessing the K2 method individually and the file with the assignment of reads (K2_r2lca_spTaxID.txt) was used to merge the results from the two classifiers.

### Metrics

As each sample belongs to only one species (*i*.*e*., the focal species), we assumed that *all* reads belong to this species. However, this is not exactly true, because the samples also contain exogenous DNA that is also sequenced (*e*.*g*., gut content, parasites, food, etc.). Nevertheless, we classified the reads into three categories: true positive (TP, when the read was assigned to the focal species), false positive (FP, when the read was assigned to a different species) and false negative (FN). The consideration of a read as a false negative is tricky, because, in addition to the exogenous DNA mentioned above, most of the reads correspond to nuclear DNA and, therefore, will not map into the mitogenomes. Thus, here we declared as a false negative a read not assigned to any species by one classifier but assigned to the focal species by the other classifier. As an example, let’s consider a read *r* assigned to the focal species by BM and not assigned (NA) by K2; this read *r* would be labelled as TP by BM and as FN by K2.

The true negative category (TN, when a read did not belong to any species was not assigned) is omitted, because all DNA sequences may be originated from a specimen (either from the focal species or exogenous DNA); one may argue that not assigned artefactual reads (*e*.*g*., chimeric reads or reads loaded with sequencing errors) belong to this group, but we cannot distinguish them from not assigned reads due to database incompleteness. So, we ignore the true negatives in the analysis.

We used the following five metrics to evaluate the performance of each classifier and their combinations.

- Richness: Number of species assigned in each library.
- Relative proportion of informative reads (RPIR): proportion of assigned reads (TP + FP) over the total number of reads in the sample (after quality control).
- Precision: ratio of true positive reads to the total assigned reads (TP + FP).
- Recall: ratio of true positive reads of the assessed classifier and true positive reads by any tool (TP + FN).
- Execution time: total consumed time by running the complete pipeline.

### Software and hardware

The software used to run the pipelines was BLASTn v2.10.0 [5], MEGAN6 v6.18.11 [21] and Kraken2 v2.0.8-beta [22]. Custom software was written in python 3.7 and is available at GitHub https://github.com/LidiaGS/ensemble_BM_K2.

Pipelines were run on a cluster with twelve identical compute nodes, each with the same architecture: two AMD Opteron(tm) Processor 4180 with 6 cores each, so 12 threads per node were available.

## Supporting information

S1 Table List of assigned species for each single-species library and pipeline. The pipelines are specified as the sheet name being BM: BLASTn + MEGAN6 pipeline; K2: Kraken2 pipeline; Unions: Union of results by BM and K2; and Intersection: Intersection of results by BM and K2. Species assignments are divided in five blocks: A: species with a relative abundance greater or equal than ε = 0.01; B: species with abundance between ε = 0.01 and ε = 0.001; C: species with abundance between ε = 0.001 and ε = 0.0001; D: species with abundance below ε = 0.0001; E: potential contaminants. For each identified species, we indicate the species name and, in brackets, the number of assigned reads and their relative abundance separated by "|".
(XLSX)

S2 Table Results for metrics benchmarked in this study for all pipelines and libraries. The metrics are specified as the sheet name. First row indicates the pipeline, being BM: BLASTn + MEGAN6 pipeline; K2: Kraken2 pipeline; Union: Union of results by BM and K2; and Intersection: Intersection of results by BM and K2. Last row contains the mean and standard deviation. For metrics of richness, RPIR, recall and precision we provide three subtables, one for each tested analytical detection limit: None, 0.0001 and 0.001.
(XLSX)

S3 Table List of reference mitogenomes used in this study. For each mitogenome we indicate a unique id code, species name, taxId code from NCBI taxonomy browser, Family and Order, length of the mitogenome and source of the mitogenome (RefSeq or GenBank).
(XLSX)
